# Gait Analysis Using Floor Markers and Inertial Sensors

**DOI:** 10.3390/s120201594

**Published:** 2012-02-07

**Authors:** Tri Nhut Do, Young Soo Suh

**Affiliations:** School of Electrical Engineering, University of Ulsan, Mugeo 2-Dong, Nam gu, Ulsan City 680-749, Korea; E-Mail: trinhutdo@gmail.com

**Keywords:** gait analysis, image processing, inertial sensors, position estimation

## Abstract

In this paper, a gait analysis system which estimates step length and foot angles is proposed. A measurement unit, which consists of a camera and inertial sensors, is installed on a shoe. When the foot touches the floor, markers are recognized by the camera to obtain the current position and attitude. A simple planar marker with 4,096 different codes is used. These markers printed on paper are placed on the floor. When the foot is moving off the floor, the position and attitude are estimated using an inertial navigation algorithm. For accurate estimation, a smoother is proposed, where vision information and inertial sensor data are combined. Through experiments, it is shown that the proposed system can both track foot motion and estimate step length.

## 1. Introduction

Gait analysis [1] involves the measurement of temporal/spatial characteristics (step length and walking speed), kinematics and kinetics. Gait analysis is used for medical purposes, sport analysis, and also for entertainment purposes. An instrumented walkway, where pressure sensors are located on the floor, can be used to measure step length and foot pressure [1]. A vision-based motion tracking system can measure step length and foot angles accurately [2]. However, both systems have rather limited walking ranges (usually less than 10 m).

Recently, inertial sensor based systems are becoming popular. In [3], inertial sensors are installed on a leg and gait phases are identified by computing angles of leg segments. In [4], inertial sensors are used to estimate upper-limb orientation. More relevant results are given in [5,6], where inertial sensors are installed on a shoe or a leg and the foot movement is estimated using inertial navigation algorithms. Step length is estimated in [5] and a foot movement is estimated in [6]. These techniques can estimate step length or foot movement without range limitations. However, due to double integration errors, the accuracy tends to degrade as time goes by.

In this paper, a new gait analysis system is proposed. A measurement system consisting of a camera and inertial sensors is installed on a shoe. Fiducial markers are printed on paper and placed on the floor. Fiducial markers are used mainly in virtual reality systems and there are many types of such markers [7–9]. In this paper, we use simple markers since there are no concerns that complicated backgrounds might mistakenly be recognized as markers.

When a foot is on the floor, the position and attitude are estimated using a camera by recognizing the markers. When a foot is moving, its movement is estimated using inertial sensors. In an inertial-based foot estimation system, Kalman filters are usually used to estimate the position and attitude of the foot. For better accuracy, a smoother is used to combine vision information and inertial sensor data. It will be shown that the accuracy of the proposed system is better than that of inertial sensor-only estimation as described in [5].

One possible application of the proposed system is the clinical gait assessment of patients, where long walking ranges are desirable. Another application is parameter estimation of some pedestrian navigation algorithms [10]. In those algorithms, relationship between step length and one step walking time needs to be identified; thus step length and one step walking time should be accurately measured. We note that step length measurement using vision only [11] and partial combinations of vision and inertial sensors are reported in [12]. In this paper, vision and inertial sensor data are tightly coupled in the proposed algorithm. The proposed system can be applied only for the flat ground. For example, the proposed system cannot be used for stairs.

The paper is organized as follows. In Section 2, an overview of the proposed system is given. In Section 3, how to estimate the position and attitude is explained. In Section 4, an inertial navigation algorithm combining inertial sensor data and vision data is described. A smoothing algorithm [13] consisting of a forward filter and a backward filter is used. In Section 5, experiment results are given. Conclusions are presented in Section 6.

## 2. Gait Analysis System

**Figure 1.**
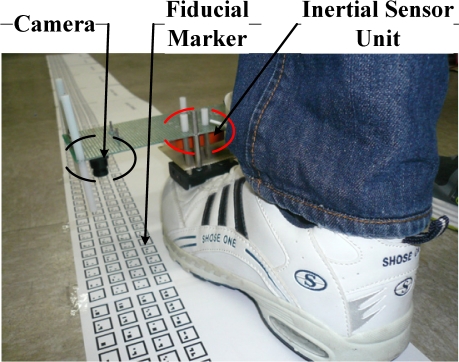
Gait Analysis System.

The gait analysis system consists of a sensor unit placed on a shoe and fiducial markers located on the floor (Figure 1). A sensor unit consists of a camera (point grey Firefly MV FFMV-03MTM) and inertial sensors (XSens MTi inertial measurement unit).

A fiducial marker used in this paper is similar to ARTag markers [7–9]. Each marker is a nine by nine grid of quadrilateral cells of 1.5 millimeter edges and thus the marker size is 13.5 mm × 13.5 mm. The intra-marker space is 4.5 mm. The marker size is determined so that there are at least two markers in the camera image. We note that the height of a camera from the markers is about 85 cm and camera field of view is about 54.5 × 45 mm.

**Figure 2.**
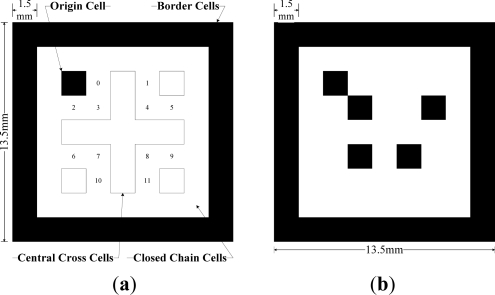
Fiducial Marker Structure (**a**) and a marker ID sample (**b**).

The whole cells are blank or colored in black in order to make them bitonal cells allowing either gray scale or color cameras to be used. The cell in black represent 1s, while the cells in white represent 0 s (Figure 2). The border cells are colored in black and are one cell width wide, followed by blank closed-chain cells. The digital coding system used to identify the marker consists of a five by five grid of bitonal cells including the blank central-cross cells, a black origin cell and three blank corner cells in the interior of a marker. A marker structure and its twelve bits digital coding system inside are illustrated in Figure 2.

The code bits are distributed along the quadrilateral central-cross in the order from left to right, and from top to bottom (the bit corresponding to 0 position in Figure 2(a) is the least significant bit and 11 is the most significant bit). The origin quadrilateral cell and three blank corner cells are used to detect the least significant bit regardless of the relative orientation between a camera and a marker. For example, the marker ID in Figure 2(b) is 000110101000 in a binary number and 424 in a decimal number.

Usually, the digital coding system is encoded in the marker containing a marker ID, checksum and error correcting codes. However, in our application, markers are seen by the camera in predetermined sequences not random sequences as in other virtual reality applications. Thus, in order to increase the recognition speed and number of markers, a simple coding system is used. Neither checksum nor error correcting codes is used. Totally, there are 2^12^=4096 unique markers (marker ID 0∼4095).With 4096 unique markers, the total length is 16.9 m. If longer walking range is needed, markers can be repeated so that marker ID 0 reappears after the marker ID 4095.

**Figure 3.**
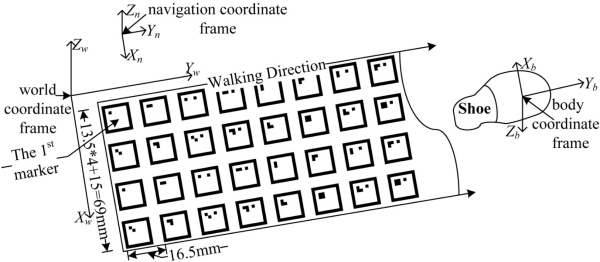
Planar Landmark System.

A planar landmark system is generated by *N* fiducial markers which are composed of a four by M grid of markers as in Figure 3. The marker ID is encoded from zero to (*N*−1).

There are four coordinate frames in this paper. The world coordinate frame is based on the marker plane: *x* and *y* axes of the world coordinate frame constitute the plane where markers are placed. The origin of the world coordinate frame coincides with the center of the first marker. Foot positions are expressed in the world coordinate frame.

The navigation coordinate frame is used in an inertial navigation algorithm. The origin of the navigation coordinate frame is the same as that of the world coordinate frame. The *z* axis of the navigation coordinate frame coincides with the local vertical. Unlike a usual navigation coordinate frame (where the *x* axis is in the direction of the magnetic north), the *x* axis lies on the *x–z* plane of the world coordinate frame. If markers are placed on the perfect plane, the inclination angle of the floor is zero and two coordinate frames are the same.

The camera coordinate frame is placed at the pinhole of the camera, where the *z* axis is perpendicular to the image plane. Three axes of the body coordinate frame coincide with those of inertial sensors. In this paper, it is assumed that the three axes of the camera coordinate frame and the body coordinate frame are the same.

**Figure 4.**
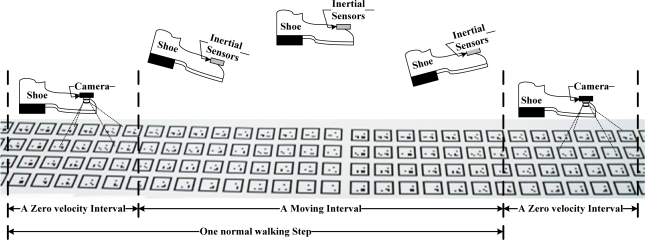
A Zero velocity interval and a moving interval of a walking step.

One walking step is illustrated in Figure 4, where normal walking is considered. When a person is walking, a foot touches the ground for a short time (usually about 0.1∼0.3 seconds) almost periodically. Even if a shoe looks like it is moving all the time during walking, a shoe is on the ground and not moving for a short time. This short interval is called the zero velocity interval. We also define moving intervals, which refer to an interval when a foot is moving. Thus one normal walking step consists of a zero velocity interval and a moving interval (see Figure 4).How to divide walking steps into a zero velocity interval and moving interval is given in Figure 4. In medical gait analysis [1], one walking step consists of seven gait phases: loading response, mid-stance, terminal stance, pre-swing, initial swing, mid-swing, terminal swing. The zero velocity interval exists between the loading response phase and terminal stance phase.

During zero velocity intervals, the position and attitude of a foot are estimated using markers on the floor (see Section 3). During moving intervals, the position and attitude are estimated using inertial sensors (see Section 4).

## 3. Position and Attitude Estimation Using Markers

**Figure 5.**
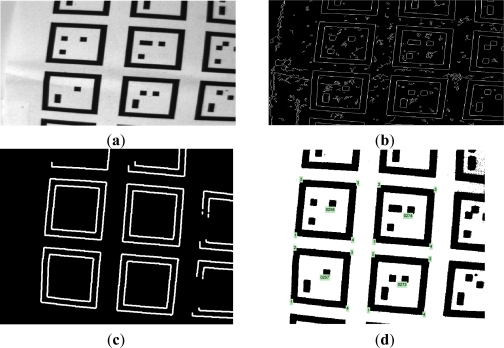
Image processing steps including (**a**) Origin grayscale image;(**b**) Detected contours using Canny edge detector;(**c**) Quadrilateral contours; and (**d**) Marker IDs and four corners.

When a foot is not moving on the floor, markers in camera images are recognized through an image processing process. The Canny algorithm, the most popular algorithm for edge detection, is applied to detect whole contours from the original captured image (Figure 5(a,b)). The quadrilateral contours are then detected based on the geometric properties (Figure 5(c)). Other contours are eliminated. Two quadrilateral contours whose central coordinates are close to each other are grouped. A line equation is computed for each side of a quadrilateral using the least squares method. For each marker, eight lines are computed (four lines for an outer quadrilateral and four lines for an inner quadrilateral). Based on these lines, the inner quadrilateral is partitioned into 7 × 7 partitions to check the interior cells (see Figure 2). The interior cells of grouped contours are converted into a binary coding using the following adaptive threshold:

$$\text{threshold}=\frac{1}{640\times 480}\left(\sum_{i=1}^{480}\left(\sum_{j=1}^{640}I_{\mathrm{ij}}\right)\right)$$

where *I_ij_* is the gray image intensity level of the (*i*, *j*) pixel. The image size is 640 × 480. If a binary coding satisfies the constraint in Figure 2, the group is identified as a marker and a marker ID is computed. If the constraint is not satisfied, the group is abandoned. From the origin cell (see Figure 2), the orientation of a marker can be identified. In Figure 5(d), identified markers with their IDs and their four corners are given, where intersections of four lines of an outer quadrilateral are defined as four corners of a marker.

Position (*r̂_vision_*) and attitude (*Ĉ_vision_*) of a camera with respect to the world coordinate frame are determined using four corners of a marker (outer corners in Figure 5(d)). Let *r_w_* be a point expressed in the world coordinate and *r_b_* be the same point in the body coordinate. Then the relationship between *r_b_* and *r_w_* is given by:

$$r_{w}={\hat{C}}_{\mathrm{vision}}r_{b}+{\hat{r}}_{\mathrm{vision}}.$$

A vector *r̂_vision_* represents the position of a camera in the world coordinate. A rotation matrix *Ĉ_vision_* represents a rotation matrix transforming a body coordinate (=camera coordinate) into a world coordinate.

It is known that the position and attitude can be computed if there are at least four points [14,15]. Thus position and attitude can be computed if there is at least one marker with four corners in the image. If there is more than one marker, more points can be used for the position and attitude computation; then the estimation error becomes smaller. We used an algorithm in [14] to estimate position *r̂_vision_* and attitude. *Ĉ_vision_* Position information is directly used in Section 4. Only yaw information in *Ĉ_vision_* is used in Section 4 since pitch and roll information can be computed from inertial sensors.

## 4. Position and Attitude Estimation Using Inertial Sensor Data

When a foot is moving, the position and attitude are estimated using an inertial navigation algorithm. To simplify the inertial navigation algorithm, it is assumed that the navigation coordinate frame and the world coordinate frame are the same. This assumption is satisfied if markers are placed on a completely flat floor.

A quaternion $q={\left[q_{0}\;\;\;\;q_{1}\;\;\;\;q_{2}\;\;\;\;q_{3}\right]}^{T}\;\in \;R^{4}$ is used to represent rotation between the navigation coordinate frame and the body coordinate frame [16,17]. The rotation matrix *C*(*q*) corresponding to quaternion *q* is defined by:

$$C\left(q\right)=\left[\begin{array}{ccc} {2q}_{0}^{2}+{2q}_{1}^{2}-1 & 2q_{1}q_{2}+2q_{0}q_{3} & {2q}_{1}q_{3}-2q_{0}q_{2}\\ 2q_{1}q_{2}-2q_{0}q_{3} & 2q_{0}^{2}+{2q}_{2}^{2}-1 & 2q_{2}q_{3}+2q_{0}q_{1}\\ 2q_{1}q_{3}+2q_{0}q_{2} & 2q_{2}q_{3}-2q_{0}q_{1} & 2q_{0}^{2}+2q_{3}^{2}-1 \end{array}\right]$$

Gyroscope output (*y_g_*) and accelerometer output (*y_a_*) are given by:

$$\begin{array}{l} y_{g}=\omega +v_{g}\\ y_{a}=C(q)\tilde{g}+a_{b}+v_{a} \end{array}$$

where *ω* is the body angular rate, *g͂* is the gravitational acceleration vector, *a_b_* is the acceleration due to movement, *v_g_* is the gyroscope measurement noise, and *v_a_* is the accelerometer measurement noise. The sensor noises *v_g_* and *v_a_* are assumed to be zero mean white Gaussian noises satisfying:

$$E[v_{g}(t)v_{g}\;{\left(s\right)}^{T}]=\lambda_{g}I\delta (t-s),\;\;\;E[v_{a}(t)v_{a}\;{\left(s\right)}^{T}]=\lambda_{a}I\delta (t-s),\;\;\;E[v_{a}(s)v_{g}\;{\left(t\right)}^{T}]=0.$$

The sampling frequency of inertial sensors is 100 Hz and the sampling frequency of vision data is 30 Hz. A discrete time system is based on the sampling period of inertial sensors; that is, the sampling period *T* for the discrete time system is *T* = 0.01 s. For example, *y_g,k_* in the discrete time means *y_g_* (*kT*) in the continuous time. Vision data is not synchronized with inertial data. When vision data is available at the continuous time *t*, its discrete time index is computed by *k* =floor(*t/T*) where floor(*U*)is the largest integer not larger than *U*.

### 4.1. Zero Velocity Interval Detection

Since the velocity of a foot cannot be measured directly, the zero velocity intervals are determined from inertial sensor data and vision data. To determine whether a discrete time *k* belongs to a zero velocity interval, firstly the following condition are tested:

(1) $$\begin{array}{cc} \left\|y_{a,i}-y_{a,i-1}\right\|<S_{a}, & \;k-\frac{N_{a}}{2}\leq i\leq k+\frac{N_{a}}{2}\\ \left\|y_{g,i}\right\|<S_{g}, & k-\frac{N_{g}}{2}\leq i\leq k+\frac{N_{g}}{2} \end{array}$$

where *S_a_*, *S_g_*, *N_a_* and *N_g_* are constant parameters. The first condition means that difference between consecutive accelerometer values should be small. The second condition means that gyroscope values should be small. In addition to Equation (1), there is an additional condition for zero velocity determination, which requires difference between two consecutive images should be small. Recall that vision data are not available for each discrete time *k* since the sampling frequency of a camera is lower than that of inertial sensor data. Here vision data means that the image coordinates of marker corners. Difference between two images is measured by computing the average movement of image coordinates of marker corners.

Given a discrete time *k*, let *k*_1_ ≤ *k* and *k*_2_ > *k* be discrete time indices at which time vision data are available. That is, vision data are available at *k*_1_ and *k*_2_ and there are no vision data in (*k*_1_,*k*_2_). Let *p*_*i,k*_1__ ∈ *R*^2^ and *p*_*i,k*_2__ ∈ *R*^2^ (1 ≤ *i* ≤ *N_k_*) be the image coordinates of marker corners, which exist both at *k*_1_ and *k*_2_ discrete time images. The same index *i* represents the same corner and *N_k_* is the number of marker corners appearing in the both images. The small image movement is defined as follows:

(2) $$\frac{1}{N_{k}}\sum_{i=1}^{N_{k}}\left\|p_{i,k_{1}}-p_{i,k_{2}}\right\|<S_{\mathrm{image}}$$

In summary, discrete time *k* is determined to belong to a zero velocity interval if Equations (1) and (2) are satisfied.

### 4.2. Initialization of an Inertial Navigation Algorithm

We use the position estimate in Section 3 as an initial position estimate. For an initial attitude estimate, we could have used the attitude estimate (*Ĉ_vision_*) from vision data. Instead we combine the attitude estimate (*Ĉ_vision_*) and accelerometer output (*y_a_*), where pitch and roll are estimated using *y_a_* and yaw is estimated from *Ĉ_vision_*. In this way, we can obtain a correct attitude estimate even if the floor is not completely flat and thus the navigation and world coordinate frames are not exactly the same.

An initial attitude estimate is computed by finding *C*(*q*) satisfying the following:

(3) $$y_{a}=C(q)\tilde{g}$$

(4) $${\hat{C}}_{\mathrm{vision}}\;\left[\begin{array}{c} 1\\ 0\\ 0 \end{array}\right]=C(q)\left[\begin{array}{c} 1\\ 0\\ 0 \end{array}\right]$$

The TRIAD algorithm [18] is used to compute *C*(*q*). We note that in the TRIAD algorithm pitch and roll are estimated using Equation (3) and yaw is estimated using Equation (4). Thus *Ĉ_vision_* only affects yaw in *C*(*q*). We have not used magnetic sensors since there could be large magnetic disturbances indoors, which degrades the accuracy of yaw estimation.

### 4.3. Basic Equations for an Inertial Navigation Algorithm

Let *v_n_* be the velocity of a foot in the navigation coordinate frame. Basic equations for *q*, *v_n_* and *r_n_* are given by:

(5) $$\begin{array}{l} \dot{q}=\frac{1}{2}\Omega (\omega )q\\ {\dot{v}}_{n}=C{\left(q\right)}^{T}\;a_{b}\\ {\dot{r}}_{n}=v_{n} \end{array}$$

where Ω(*ω*) is defined by:

$$\Omega (\omega )\;@\left[\begin{array}{cccc} 0 & -\omega_{1} & -\omega_{2} & -\omega_{3}\\ \omega_{1} & 0 & \omega_{3} & -\omega_{2}\\ \omega_{2} & -\omega_{3} & 0 & \omega_{1}\\ \omega_{3} & \omega_{2} & -\omega_{1} & 0 \end{array}\right]$$

In this paper, we estimate *q*, *v_n_* and *r_n_* by combining a forward filter and a backward filter [19]. An indirect filter is used for a forward and a backward filter. Firstly *q*, *v_n_* and *r_n_* are estimated using Equation (5); let *q̂*, *v̂_n_* and *r̂_n_* be corresponding estimated values. Let *q̃_e_*, *v_e_* and *r_e_* be errors of these estimated values, which are defined by:

$$\begin{array}{l} {\tilde{q}}_{e}={\hat{q}}^{*}\;⊗q\\ v_{e}=v_{n}-{\hat{v}}_{n}\\ r_{e}=r_{n}-{\hat{r}}_{n} \end{array}$$

where ⊗ represents the quaternion multiplication. Assuming that *q̃_e_* is small, we can approximate *q̃_e_* as follows:

$${\tilde{q}}_{e}=\left[\begin{array}{c} 1\\ q_{e} \end{array}\right]$$

where *q_e_* ∈ *R*^3^. In an indirect filter, *q_e_*, *v_e_* and *r_e_* are estimated. Let a state *x* be defined by:

$$x=\left[\begin{array}{c} q_{e}\\ v_{e}\\ r_{e} \end{array}\right]\in R^{9}$$

The differential equation is given by:

(6) $$\dot{x}(t)=A(t)x(t)+w(t)$$

where:

$$A=\left[\begin{array}{ccc} -\left[y_{g}\times \right] & 0 & 0\\ -2C\;{\left(\hat{q}\right)}^{T}\;\left[y_{a}\times \right] & 0 & 0\\ 0 & I & 0 \end{array}\right],\;\;\;\;\;\;\;\;\;\;\;\;\;\;\;w=\left[\begin{array}{c} -0.5v_{g}\\ -C{\left(\hat{q}\right)}^{T}\;\eta_{a}\\ 0 \end{array}\right].$$

For a vector *p* ∈ *R*^3^, [*p*×] is defined by:

$$p=\left[\begin{array}{ccc} 0 & -p_{3} & -p_{2}\\ p_{3} & 0 & -p_{1}\\ -p_{2} & p_{1} & 0 \end{array}\right].$$

Note that E[*w*(*t*)*w*(*t*)*^T^*]=*Qδ*(0), where:

$$Q=\left[\begin{array}{ccc} 0.25\lambda_{g}I & 0 & 0\\ 0 & \lambda_{a}I & 0\\ 0 & 0 & 0 \end{array}\right].$$

### 4.4. Forward Filter

A forward filter processes data from the beginning to the end of data. In a forward filter, *q̂_f,k_*, *v̂_f,k_* and *r̂_f,k_* are first computed using the discretized approximation of Equation (5): subscript *f* represents a forward filter and *k* represents the discrete time index. The following equations are used to compute *q̂_f,k_*, *v̂_f,k_* and *r̂_f,k_*:

$$\begin{array}{l} {\hat{q}}_{f,k+1}=\left(I+\frac{3}{4}\Omega_{k}T-\frac{1}{4}\Omega_{k-1}T-\frac{1}{6}{\left\|y_{g,k}\right\|}_{2}^{2}T^{2}-\frac{1}{24}\Omega_{k}\Omega_{k-1}T^{2}-\frac{1}{48}{\left\|y_{g,k}\right\|}_{2}^{2}\Omega_{k}T^{3}\right){\hat{q}}_{f,k}\\ {\hat{v}}_{f,k+1}={\hat{v}}_{f,k}+0.5T\left(C{\left({\hat{q}}_{f,k+1}\right)}^{T}\;y_{a,k+1}+C{\left({\hat{q}}_{f,k}\right)}^{T}\;y_{a,k}\right)-T\tilde{g}\\ \;\;\;\;\;\;\;\;\;\;\;\;\;\;\;\;\;\;\;\;\;\;\;\;\;\;\;\;\;\;\;\;\;\;\;\;\;\;\;\;\;\;\;\;\;\;\;\;{\hat{r}}_{f,k+1}={\hat{r}}_{f,k}+0.5T\left({\hat{v}}_{f,k+1}+{\hat{v}}_{f,k}\right)\; \end{array}$$

where Ω*_k_* is defined by Ω*_k_* = Ω(*y_g,k_*). In the computation, *q̂_f,k_* is normalized each time.

The estimation errors in *q̂_f,k_*, *v̂_f,k_* and *r̂_f,k_* are estimated using a forward Kalman filter, where the process model is given in Equation (6). During the zero velocity interval, the fact *v_n,k_* = 0 is used in the measurement equation. If vision data are also available, *r̂_vision,k_* and *Ĉ_vision_* are used in the measurement update. For example, a measurement equation using *v_n,k_* = 0 and *r̂_vision,k_* is given by:

$$z_{k}=\left[\begin{array}{c} 0-{\hat{v}}_{f,k}\\ {\hat{r}}_{\mathrm{vision},k}-{\hat{r}}_{f,k} \end{array}\right]=\left[\begin{array}{ccc} 0 & I & 0\\ 0 & 0 & I \end{array}\right]x_{k}+v_{k}.$$

The measurement noise *v_k_* is assumed to satisfy:

$$E[v_{k}v_{k}{\;}^{T}]=\left[\begin{array}{cc} r_{v}I & 0\\ 0 & r_{r}I \end{array}\right]$$

where *r_v_* and *r_r_* are scalar constants. Similarly to the initialization process, only yaw information in *Ĉ_vision_* is used. We used the same technique in [20] for yaw updating. In [20], magnetic sensors are used for yaw updating. Replacing the magnetic sensor outputs with *Ĉ_vision_* [1 0 0]*^T^*, we can obtain a yaw updating algorithm.

The error covariance of the forward Kalman filter is denoted by *P_f,k_* :

$$P_{f,k}=E[(x_{f,k}-{\hat{x}}_{f,k}){\left(x_{f,k}-{\hat{x}}_{f,k}\right)}^{T}]\in R^{9\times 9}.$$

### 4.5. Backward Filter

A backward filter processes data from the end of data to the beginning. Similarly to the forward filter, *q̂_b,k_*, *v̂_b,k_* and *r̂_b,k_* are first computed (subscript *b* represents a backward filter) :

$$\begin{array}{l} {\hat{q}}_{b,k}=\left(I-\frac{3}{4}\Omega_{k}T+\frac{1}{4}\Omega_{k+1}T-\frac{1}{6}{\left\|y_{g,k}\right\|}_{2}^{2}T^{2}-\frac{1}{24}\Omega_{k}\Omega_{k+1}T^{2}+\frac{1}{48}{\left\|y_{g,k}\right\|}_{2}^{2}\Omega_{k}T^{3}\right){\hat{q}}_{b,k+1}\\ {\hat{v}}_{b,k}={\hat{v}}_{b,k+1}+0.5T\;\left(C{\left({\hat{q}}_{b,k+1}\right)}^{T}\;y_{a,k+1}+C{\left({\hat{q}}_{b,k}\right)}^{T}\;y_{a,k}\right)-T\tilde{g}\\ \;\;\;\;\;\;\;\;\;\;\;\;\;\;\;\;\;\;\;\;\;\;\;\;\;\;\;\;\;\;\;\;\;\;\;\;\;\;\;\;\;\;\;\;\;\;\;\;{\hat{r}}_{b,k}={\hat{r}}_{b,k+1}+0.5T\;\left({\hat{v}}_{b,k+1}+{\hat{v}}_{b,k}\right) \end{array}$$

Their errors are estimated in the backward filter, where the state space model is given by:

$${\dot{x}}_{b}=-{\mathrm{Ax}}_{b}-w$$

where the state definition of *x_b_* is the same as that of *x_f_*.

The measurement equations are the same as those in the forward filter. The error covariance of the backward Kalman filter is denoted by *P_b,k_*:

$$P_{b,k}=E[(x_{b,k}-{\hat{x}}_{b,k}){\left(x_{b,k}-{\hat{x}}_{b,k}\right)}^{T}]\in R^{9\times 9}$$

### 4.6. Smoother

The smoother combines the forward filter output and the backward filter output. Due to an indirect filter structure, it is difficult to derive an optimal smoother. In this paper, we use a suboptimal smoother, which only uses diagonal matrices of *P_f,k_* and *P_b,k_*.

Diagonal matrices of *P_f,k_* and *P_b,k_* are given by:

$$P_{f,k}=\left[\begin{array}{ccc} P_{q_{f},k} & * & *\\ {}* & P_{v_{f},k} & *\\ {}* & * & P_{r_{f},k} \end{array}\right],\;\;P_{b,k}=\left[\begin{array}{ccc} P_{q_{b},k} & * & *\\ {}* & P_{v_{b},k} & *\\ {}* & * & P_{r_{b},k} \end{array}\right]$$

where * parts denote the off-diagonal matrices and not used in the smoother. The smoothed quaternion is computed by using the quaternion averaging algorithm in [21]. The smoothed quaternion *q_s_* is computed by solving the following optimization problem:

$${\hat{q}}_{s,k}={\text{min}}_{q}\;q^{T}\left(\Xi {\left(q_{f,k}\right)}^{T}\;P_{q_{f},k}{}^{-1}\Xi (q_{f,k})+\Xi {\left(q_{b,k}\right)}^{T}\;P_{q_{b},k}{}^{-1}\Xi (q_{b,k})\right)q$$

where:

$$\Xi (q)=\left[\begin{array}{cccc} -q_{1} & q_{0} & -q_{3} & q_{2}\\ -q_{2} & q_{3} & q_{0} & -q_{1}\\ -q_{3} & -q_{2} & q_{1} & q_{0} \end{array}\right]$$

The smoothed velocity and position estimates (*v̂_s_* and *r̂_s_*) are given by:

$$\begin{array}{l} {\hat{v}}_{s,k}={\left(P_{v_{f},k}{}^{-1}+P_{v_{b},k}{}^{-1}\right)}^{-1}\;(P_{v_{f},k}{}^{-1}{\hat{v}}_{f,k}+P_{v_{b},k}{}^{-1}{\hat{v}}_{b,k})\\ {\hat{r}}_{s,k}={\left(P_{r_{f},k}{}^{-1}+P_{r_{b},k}{}^{-1}\right)}^{-1}(P_{r_{f},k}{}^{-1}{\hat{r}}_{f,k}+P_{r_{b},k}{}^{-1}{\hat{r}}_{b,k}). \end{array}$$

## 5. Experimental Results

In this section, experimental results to verify the proposed method are given. The camera is calibrated to obtain its intrinsic parameters [22]. The inertial sensor unit is calibrated using the algorithm in [23]. The sampling rate of camera and inertial IMU sensors are 30 fps and 100 Hz, respectively. The computation is done off-line using Matlab. The thresholds used for zero velocity detection are given as follows:

$$N_{a}=0.1,\;S_{a}=20,\;N_{g}=0.05,\;S_{g}=20,\;S_{\mathrm{image}}=30$$

where these thresholds are decided by trial and error. Various combinations of thresholds are tried and the above thresholds are chosen since they identify zero velocity intervals well. In this process, true zero velocity intervals are manually identified by inspecting inertial sensor data.

The initial values used for the indirect Kalman filter in the experiment are given as follows:

$$\begin{array}{c} P_{q_{f},0}=P_{q_{b},0}=0.001I,\;P_{v_{f},0}=P_{v_{b},0}=0.001I,\;P_{r_{f},0}=P_{r_{b},0}=0.008I\\ r_{v}=0.004,\;r_{r}=0.008,\;R_{\mathrm{yaw}}=0.01I \end{array}$$

where *P*_*q_f_*,0_, *P*_*v_f_*,0_, *P*_*r_f_*,0_ are the initial values of forward Kalman filter error covariance matrices, *P*_*q_b_*,0_, *P*_*v_b_*,0_, *P*_*r_b_*,0_ are the initial values of backward Kalman filter error covariance matrices, respectively.

### 5.1. Table Experiment: Comparison with the Digitizer Output

**Figure 6.**
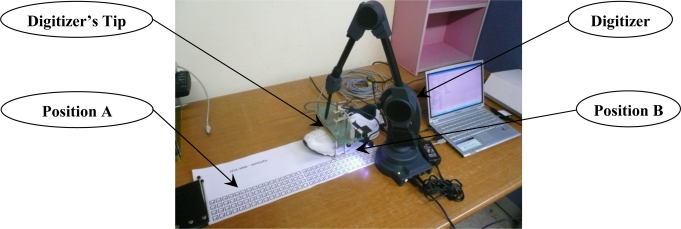
Table experiment (*Y_w_* axis moving experiment).

A shoe is moved back and forth between two positions A and B several times along to the *Y_w_* axis direction as illustration in Figure 6. The movement is tracked using Microscribe G2X digitizer, whose output is considered as a true value. The accuracy of G2X model is up to 0.23 mm in a 1.27m sphere workspace.

**Figure 7.**
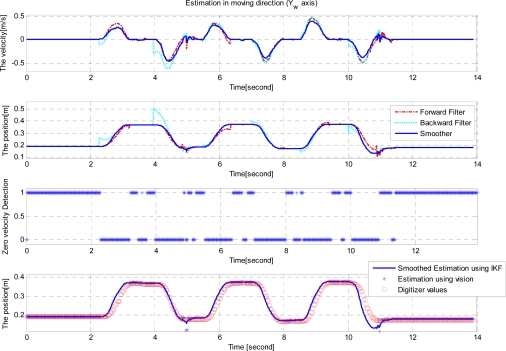
Results of a table experiment (*Y_w_* axis moving experiment).

Since the movement is mainly along the *Y_w_* axis, only the *Y_w_* axis velocity and position are plotted in Figure 7. The first graph of Figure 7 shows the estimated velocity using the forward filter, the backward filter and the smoother while the second graph shows the position estimates. The third graph shows the zero velocity detection results, where the value 1 indicates that the corresponding discrete time belong to a zero velocity interval. In the second graph, assuming the smoother estimates are accurate, we can see the error in the forward filter increases as the moving time increases. Also we can see the error in the backward filter increases as the moving time backwardly increases. In the final graph of Figure 7, *Y_w_* axis position estimated by the smoother, the vision, and the digitizer are given. The RMS difference between digitizer data and smoothed estimation is 4.8 ± 9.1 mm (mean ± standard deviation).

### 5.2. Walking Experimental Results

**Figure 8.**
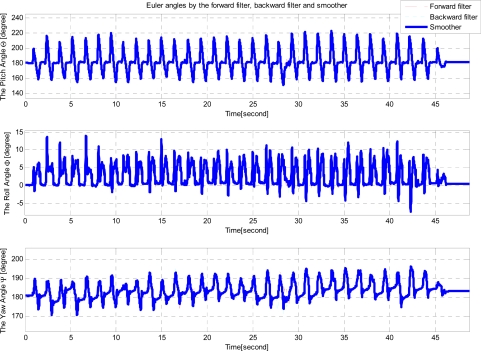
Floor experiment (Euler angles estimation).

A person wearing the shoe walked along a planar marker system path (Figure 1). The length of the path is 33.8 meters. We note that this length can be easily extended by using more markers. Euler angles of a shoe are given in Figure 8.

**Figure 9.**
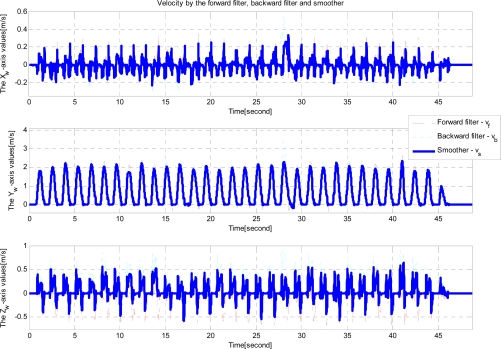
Floor experiment (velocity estimation).

**Figure 10.**
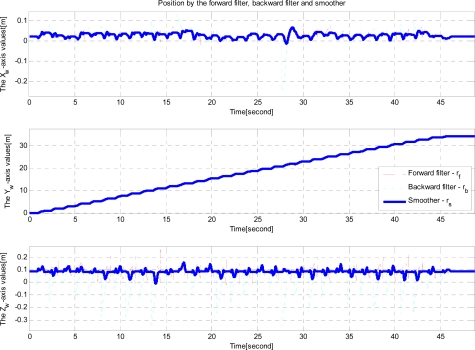
Floor experiment (position estimation).

Note that quaternion is used to represent attitude. Euler angles are transformed from quaternion estimates for visualization. In the attitude estimation, there are almost no differences between the forward and backward filter estimates. This is due to the fact that attitude errors are almost periodically reset during zero velocity intervals. Velocity and position estimation results are given in Figures 9 and 10.

Note that there are large differences between the forward and backward filters. The errors are probably due to inertial sensor errors (bias and scaling factor). We can see the velocity and position estimation errors can be reduced by using the smoother.

**Figure 11.**
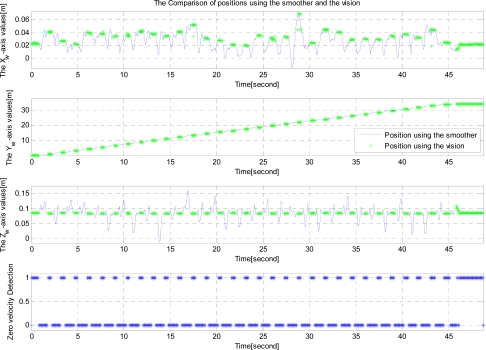
Floor experiment (Comparison of smoother estimated and vision estimated positions).

The position estimates from vision is compared with smoothed position estimates in Figure 11. Note that the position estimated from vision is mostly available during zero velocity intervals. During moving intervals, marker recognition becomes difficult due to motion related image blurring.

**Figure 12.**
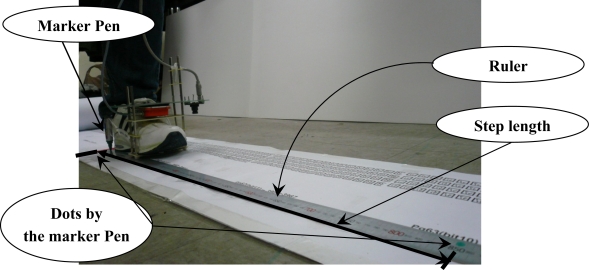
One walking step length measurement.

From Figure 10, step length can be computed. One walking step consists of a zero velocity interval and a moving interval (see Figure 4). The accuracy of the step length estimation is evaluated by one-step experiment using the ruler as a reference (see Figure 12). A marker pen is attached on the shoe. Its tip touches on the ruler when the shoe is on the ground. Step length measured by the ruler (by measuring two dots) is considered as a true value.

The results of 20 one-step experiments are listed in Table 1. The error between the measurement using a ruler and the smoothed estimation is in range 0.5∼4.1 mm. RMS difference and the worst case error are given in Table 2.

The RMS error between the measurement by a ruler and the smoothed estimation is 1.99 ± 1.25 mm (mean ± standard deviation). They are smaller than the one (4.8 ± 9.1 mm) in Section 5.1 since the step length is computed using estimates during zero velocity intervals. During zero velocity intervals, positions are compensated from the vision and thus position estimates are more accurate than those during moving intervals. Step length RMS error in [5] is 34.1 ± 2.1 mm, where an optical tracker output is used as a true value. The proposed system is more accurate since markers are used to compensate position and attitude errors.

## 6. Conclusions

A gait analysis system combining a reliable fiducial marker system on the floor and a inertial sensor unit was proposed. The system can track foot motion and measure step length on flat ground. The position errors tend to become larger during the moving intervals and smaller during the zero velocity intervals since vision data are used to reduce position and attitude errors. The step length RMS error is 1.99 ± 1.25 mm, which is smaller than that of an existing inertial sensor only system. Commercial optical trackers such as the Vicon unit is more accurate than the proposed system—they are usually sub-millimeter level, however, they have rather limited walking ranges. On the other hand, the walking range of the proposed system can be easily extended.

## Figures and Tables

**Table 1.** Step length estimation (20 steps).

	**Step #1**	**Step #2**	**Step #3**	**…**	**Step #18**	**Step #19**	**Step #20**
**Measurement by a ruler [mm]**	792	804	841	…	779	800	814
**Estimation by a smoother [mm]**	793.6	802.2	839.6	…	778.5	797.8	810.0
**Error [mm]**	1.6	1.8	1.4	…	0.5	2.2	4.0

**Table 2.** Step length estimation error.

**Mean of step length error [mm]**	1.99
**Standard deviation of step length error [mm]**	1.25
**Maximum value of step length error [mm]**	4.10
